# The efficacy of adding group behavioral activation to usual care in patients with fibromyalgia and major depression: design and protocol for a randomized clinical trial

**DOI:** 10.1186/s13063-018-3037-1

**Published:** 2018-11-29

**Authors:** Lydia Gómez-Pérez, Alvaro Vergés, Ana Rocío Vázquez-Taboada, Josefina Durán, Matías González Tugas

**Affiliations:** 1 Escuela de Psicología. Facultad de Ciencias Sociales, Pontificia Universidad Católica de Chile, Campus San Joaquín. Avda. Vicuña Mackenna 4860, Macul Santiago, Chile; 2 Departamento de Reumatología, Escuela de Medicina, Pontificia Universidad Católica de Chile, Avda. Libertador Bernardo O’Higgins, 340 Santiago, Chile; 3 Departamento de Psiquiatría, Escuela de Medicina, Pontificia Universidad Católica de Chile, Avda. Libertador Bernardo O’Higgins 340, Santiago, Chile

**Keywords:** Fibromyalgia, Depression, Behavioral activation, Group intervention, Efficacy, Chile

## Abstract

**Background:**

Fibromyalgia and major depression frequently co-occur. Patients with both conditions have a worse prognosis and higher disability, and their treatment options are scarce. Behavioral activation (BA) may be an especially useful intervention for these patients, as it targets mechanisms of action that seem to be common to both disorders. Nevertheless, its efficacy has not been examined in people with both conditions. We describe the design and rationale of a randomized clinical trial aimed to evaluate the efficacy of adding BA (applied in groups) to usual care in order to reduce the severity of depressive symptoms (primary outcome) among Chilean women with fibromyalgia and major depression (*N* = 90). Pain intensity, fibromyalgia impact, pain catastrophizing and hypervigilance, physical health symptoms, environmental reward, and BA will be evaluated as secondary outcomes.

**Methods:**

Women will be randomized to an experimental arm (*n* = 45) which will receive usual care (UC) for fibromyalgia with comorbid depression plus BA; and a comparison arm, which will receive only UC for fibromyalgia with comorbid depression (n = 45). Outcome assessment will take place at four time points: (1) at baseline, (2) when the experimental arm is under treatment (between sessions 6 and 7), (3) immediately after the experimental arm complete the treatment, and (4) at a 3-month follow-up. The following instruments will be used: Chilean version of the Patient Health Questionnaire-9 (PHQ-9*),* Composed Pain Intensity Index, Fibromyalgia Impact Questionnaire Revised (FIQ-R), Pain Catastrophizing Scale (PCS), Pain Vigilance and Awareness Questionnaire (PVAQ), Patient Health Questionnaire (PHQ-15), Reward Probability Index (RPI), and the Activation subscale of the Behavioral Activation for Depression Scale (BADS).

**Discussion:**

We expect that, after treatment, the group receiving BA should experience greater reductions in the primary and secondary outcomes than the group receiving only UC. These reductions should be both statistically and clinically significant and will be maintained at follow-up. This study will contribute to facilitate the integrated treatment of fibromyalgia and depression.

**Trial registration:**

ClinicalTrials.gov under the name “Testing Interventions for Patients with Fibromyalgia and Depression,” Identifier: NCT03207828. Registered on 5 July 2017 (last update posted 21 September 2017).

**Electronic supplementary material:**

The online version of this article (10.1186/s13063-018-3037-1) contains supplementary material, which is available to authorized users.

## Background

Fibromyalgia is a common, chronic, widespread pain disorder that seriously affects quality of life. Treatment of fibromyalgia is an unsolved issue, with multiple existing treatments that have limited efficacy. Between 62 and 86% of patients with fibromyalgia suffer from major depression and 90% from depressive symptomatology [1]. As such, Gracely, Ceko, and Bushnell (2012) posit that fibromyalgia and depression are two manifestations of a single affective spectrum disorder [1]. In fact, according to some evidence, both disorders share pathophysiological aspects [2]. Furthermore, depressive symptoms are associated with a worse pain prognosis [3]. Patients with fibromyalgia typically first present and seek continued treatment in primary care, representing an important subpopulation in that context [4]. The increasing and concomitant prevalence of depression and fibromyalgia represents a burden for primary care systems, and providing effective and appropriate primary care interventions to address this co-occurrence is indeed needed.

Behavioral activation (BA) is an evidence-based therapy for depression [5, 6]. It has been shown to be as effective as comprehensive cognitive behavioral therapy [5, 7] and antidepressant therapy [8], with a lower dropout rate than the latter [8]. In addition, BA has been shown to work as effectively as medication and better than cognitive behavioral therapy in the treatment of severe depression [8]. Several meta-analyses support its efficacy [5, 9, 10] and it is much easier to apply and disseminate than other therapies [6].

Because BA focuses on fighting inactivity, increasing environmental reinforcers, and decreasing aversive experiences, it could be particularly suitable for treating depression among patients with fibromyalgia, as these factors seem to play a key role in the etiology of both disorders [6, 11]. Preliminary results have shown that BA could break the chain of perpetuation in these disorders and that it may also reduce pain intensity and pain-related anxiety and catastrophism [12, 13]. Nevertheless, to our knowledge, beyond two case studies [12, 13], no studies have been conducted to examine the efficacy of BA to reduce depressive symptoms in patients with fibromyalgia. We describe the design and rationale of a randomized clinical trial aimed to examine the efficacy of adding BA to usual care (UC) in order to reduce the severity of depressive symptoms (primary outcome) among Chilean women with fibromyalgia and comorbid major depression. The following secondary outcomes will also be examined: pain intensity, fibromyalgia impact, pain catastrophizing and hypervigilance, physical health symptoms, environmental reward, and BA.

We expect to find that, after treatment, women in the experimental arm should experience a greater decrease in depressive symptom severity as well as in several pain-related variables than women in the comparison arm. We expect that decreases observed in the pain-related variables should be mediated by decreases observed in depressive symptom severity. Outcome differences among the groups will be maintained after a 3-month follow-up.

## Methods

### Overview of the study design

**Fig. 1:**
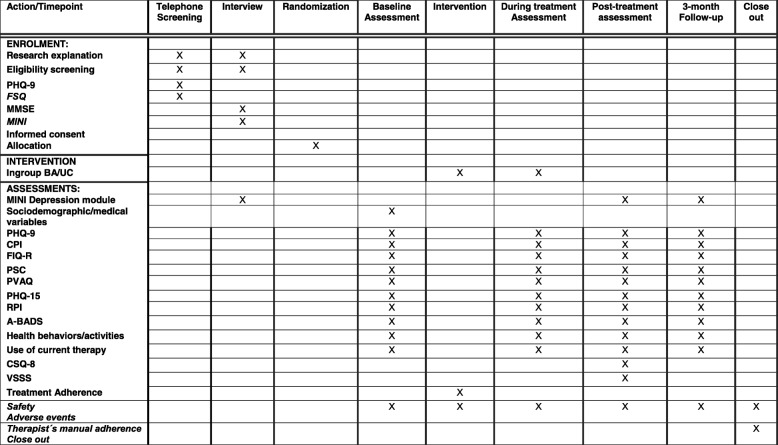
Schedule of enrollment, interventions, and assessment. Abbreviations: *PHQ-9* Patient Health Questionnaire-9, *FSQ* Fibromyalgia Survey Questionnaire, *MMSE* Mini-Mental State Examination, *MINI* Mini International Neuropsychiatric Interview, *BA* Behavioral activation, *UC* usual care, *CPI* Composite Pain Index, *FIQ-R* Fibromyalgia Impact Questionnaire, *PCS* Pain Catastrophizing Scale, *PVAQ* Pain Vigilance and Awareness Questionnaire, *PHQ-15* The Patient Health Questionnaire, *RPI* Reward Probability Index, *BADS* Activation subscale of the Behavioral Activation for Depression Scale, *CSQ-8* Client Satisfaction Questionnaire-8, *VSSS* Verona Service Satisfaction Scale

The present study is a randomized clinical superiority trial with a parallel design. Women with fibromyalgia and major depression will be randomized to either of two intervention arms: an experimental arm – that will be treated with BA in a group setting in addition to UC – and a comparison arm that will continue receiving only its UC. The comparison between these two groups will allow us to establish the potential benefits of adding group BA to the patient’s UC. Outcomes will be assessed in both groups before, during, and after the intervention, as well as after a 3-month follow-up, by a research assistant who will be blind to group assignment. The schedule of enrollment, interventions, and assessment is presented in Fig. 1 (see also Additional file 1: Standard Protocol Items: Recommendations for Interventional Trails (SPIRIT) Checklist).

#### Trial setting

Participants will be interviewed in Centro Médico San Joaquín, a university medical center that is part of the Red Salud UC CHRISTUS. The intervention will take place in the Innovation Center which is located in a campus at Pontificia Universidad Católica de Chile.

### Participants

**Fig. 2:**
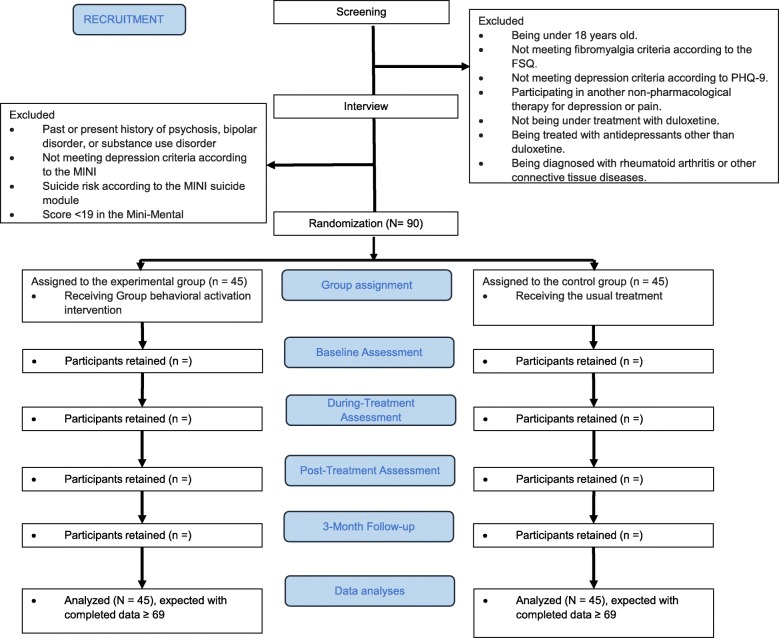
Flowchart of the research according to the Consolidated Standards of Reporting Trials (CONSORT) model

The flowchart of the research according to the Consolidated Standards of Reporting Trials (CONSORT) model is presented in Fig. 2. Women diagnosed with fibromyalgia and major depression (*N* = 90) will participate. Inclusion criteria are (1) being 18 years old or older, (2) understanding Spanish, (3) meeting the diagnostic criteria for fibromyalgia according to the Fibromyalgia Survey Questionnaire (FSQ) [14, 15], (4) having a primary diagnosis of major depression according to the Mini International Neuropsychiatric Interview (MINI) [16, 17], and (5) have being receiving UC for fibromyalgia and depression with duloxetine for at least 3 months. Exclusion criteria are (1) having a past or present history of psychosis, bipolar disorder, or substance use disorder, (2) presenting risk of imminent suicide according to the suicide module of the MINI, (3) presenting a lower score than the cut-off point in the Mini-Mental State Examination (MMSE) [18, 19], (4) to currently receive treatment with a psychologist or a psychiatrist, (5) being treated with antidepressants other than duloxetine, and (6) being diagnosed with rheumatoid arthritis or other connective tissue diseases.

#### Sample size calculation

The study sample size was calculated for a Hierarchical Linear Model (HLM) in which the primary outcome (depressive symptom severity) was the dependent variable. Using Mplus [20], we determined that a sample of 90 participants will be needed to reach a power of .80. In this power analysis, the correlation between the intercept and the slope was specified to be − 0.4. The average slope was assumed to be negative, as depression scores are expected to drop, and was specified as − 0.5. To simplify the model, the variances of the intercept, slope, and scores of all the variables in the model (depressive symptom severity, and covariates) were specified as 1, so as to have the standardized model solution. The treatment effect on the slope (standardized Beta) was specified as 0.45. This corresponds to a medium-to-large effect size [21, 22]. In addition, the effect of two covariates was included. The effect of each of the covariates on the slope was specified as 0.1. The correlation between treatment and each covariate was specified as 0.1, and the correlation between covariates was specified as 0.3.

### Recruitment

Participants will be recruited through several procedures. First, posters and brochures with information about the study will be available at the clinic rooms of the Red Salud UC CHRISTUS, as well as in the waiting room, so that patients interested in participating could contact the research team by email or telephone. In addition, one of the nurses of the medical center will contact by telephone all the fibromyalgia patients registered at the Red Salud UC CHRISTUS to ask them for permission to put them in contact with the study research team. Participants who agree to be contacted will receive a telephone call from a research assistant, who will provide them with further information about the study. Patients who agree to participate will first be interviewed by telephone and then will be invited to come to the medical center for a face-to-face interview.

#### Telephone interview

Participants will be asked whether they (1) are 18 years old or older (inclusion criteria 1). The diagnosis of fibromyalgia will be confirmed using the Fibromyalgia Survey Questionnaire. (FSQ) [14, 15]. The FSQ assesses the main symptoms of fibromyalgia according to the American College of Rheumatology. It is comprised by the Widespread Pain Index (WPI), which assesses the number of areas with pain in the last week from a total of 19 body areas; and a modified version of the Symptoms Severity Scale, which assesses tiredness, attentional or concentration problems, and waking up feeling restless in a scale ranging from 0 to 3; and includes questions regarding other diagnostic criteria present in the last 3 months. To meet a diagnosis of fibromyalgia, the patient must report (1) WPI scores ≥ 7 and symptoms severity ≥ 5 or WPI between 3 and 6 and symptoms severity ≥ 9; (2) presence of symptoms of the same level for at least 3 months; and (3) absence of other disorders that could explain the symptoms. The psychometric properties of the Spanish version of this scale have been shown to be adequate [15]. The FSQ was previously validated by the research team in a sample of Chilean women with fibromyalgia (*N* = 114) (unpublished results). Participants will also complete the Chilean version of the Patient Health Questionnaire-9 (PHQ-9*)* [23, 24]*.* It consists of nine items evaluating the presence of depressive symptoms in the last 2 weeks. Item response options are: 0 = never, 1 = some days, 2 = more than half the days and 3 = almost every day. Patients can be classified into: (1) major depressive syndrome (presence of five or more of the nine depressive symptoms with a severity index of more than half of the days, and one of the symptoms is depressive mood or anhedonia), (2) other depressive syndrome (presence of two, three or four depressive symptoms for more than half the days and one of the symptoms is depressive mood or anhedonia), and (3) positive depressive symptoms (presence of at least one or two of the depressive symptoms, but fails to complete the above criteria). The Chilean version of the PHQ-9 has shown a sensitivity of 92% and specificity of 89% [23, 24]. Finally, participants will be asked whether they (1) have been under treatment with duloxetine for at least 3 months (inclusion criteria 6), (2) are receiving treatment from a psychologist or a psychiatrist (exclusion criteria 4), (3) are being treated with antidepressants other than duloxetine (exclusion criteria 5), and (4) have been diagnosed with rheumatoid arthritis or other connective tissue diseases (exclusion criteria 6). Participants who meet the criteria assessed during this interview will be invited to a face-to-face interview.

#### Face-to-face interview

During this interview, the diagnosis of major depression will be confirmed using the MINI [16, 17]. In addition, this instrument will be used to assess the presence of disorders that are considered among the exclusion criteria (i.e., having past or present history of psychosis, bipolar disorder, or substance use disorder), and to check that participants are not at risk for imminent suicide. The MINI is a widely used, brief, structural diagnostic interview to assess the main mental disorders included in the *Diagnostic and Statistical Manual of Mental Disorders, version 4* (DSM-IV) and the *International Classification of Diseases, 10th edition* (ICD-10). It has been shown to be highly valid and reliable [16, 17]. Finally, the MMSE [18, 19] will be used to assess cognitive abilities. It comprises six items: temporal orientation, immediate recall of three words, attention or calculus (backward subtraction), differed recall of the three words, understanding one verbal order and copying a diagram. There are normative scores for Chilean population of different ages and education levels [19]. We will use a cut-off score of 19.

### Interventions

#### Control arm

When entering the study, this group of participants will be receiving UC for fibromyalgia with co-occurring depression. This involves treatment with duloxetine for at least 3 months before entering the study. UC may include physical activity, pregabalin, pain killers, and muscle relaxants in addition to duloxetine. During their participation in the trial, participants in the control arm will continue the treatment with duloxetine and additional pain medication as part of their UC.

#### Experimental arm

When entering the study, this group will be receiving UC for fibromyalgia with co-occurring depression (similar to the control group). In addition to this treatment, participants in this arm will start to receive in-group BA. This intervention is based on the Brief Behavioral Activation Treatment for Depression [21–25], whose aims are (1) to increase engagement with activities associated with pleasure or mastery experiences; (2) to decrease engagement with activities that maintain or increase the risk of depression; and (3) and to remove the barriers that limit the access to gratification. The sessions are well structured and described in the manual. The protocol was originally designed to be applied individually with patients diagnosed with major depression. However, we introduced some modifications so that it can be used with patients diagnosed with fibromyalgia. In particular, a rationale about the relationship between pain and depression was added to the first session. During this session the importance of treating depression in patients with fibromyalgia is highlighted and pain experiences are validated. Furthermore, sessions were simplified so that they can be better followed by participants with cognitive symptoms of fibromyalgia, and a treatment outlet with a summary of the main ideas discussed at each session was created. Finally, the manual has been adjusted to be applied in group sessions (five to eight participants). Core aspects of treatment were not altered. The treatment will last for 10 sessions over 2 months. Two psychologists trained in BA will lead the sessions.

### Randomization

Participants who meet all the inclusion criteria after the eligibility interview will be randomized to the two arms, with a 1:1 allocation ratio. We plan to randomize every time we are able to recruit enough participants for a treatment group and a control group. That is, randomization will take place with 16 participants each time, eight of them being assigned to the experimental arm and the other eight to the control. Nonetheless, due to recruitment and logistics issues, it is possible that sometimes a smaller or bigger group of participants needs to be randomized into the two arms. Randomization will be conducted by an independent researcher (not involved in the assessments, treatment, or data analysis). The project coordinator will submit an email to this independent researcher with the ID numbers of participants to be randomized. This researcher will use a computer program to randomly assign participants to the control and the experimental groups. The researcher will then submit a pdf file to the research coordinator with the assignment and will keep a copy for record. The project coordinator will be the only person from the team who is aware of participant allocation, and will keep this information in a file in her computer protected with a password. The principal investigator, co-investigators, and statistician will be blind to participant allocation. The project coordinator will then contact participants to inform them about the initiation of treatment. Notably, all participants will receive the BA intervention at some point. The only difference between the experimental and control groups will be that the former will receive treatment before participants in the control group. To minimize bias, all participants will be informed that they will wait before receiving the intervention and that they will be assessed periodically until their treatment begins, but they will not be aware of how long they or the other people in the study will have to wait. They will also be informed that nobody will wait more than 6 months.

## Data collection

Outcome data will be collected using standardized questionnaires, that will be administered by telephone as a structured interview by several research assistants. Outcome assessment will take place at four time points in both arms: (1) at baseline, (2) when the experimental arm is under treatment (between sessions 6 and 7), (3) immediately after the experimental arm complete the treatment, and (4) at a 3-month follow-up. As such, participants will be assessed during 5 months. Participants who drop out from the intervention will still be contacted for follow-up assessments. Research assistants will be blind to the aims and hypotheses of the study. As a compensation, patients will receive a gift card (with a value of 10,000 Chilean pesos) after each outcome assessment interview.

### Baselines and follow-up interviews

The variables assessed and the instruments used during the baseline and follow-up interviews – including the sociodemographic and medical variables, the primary outcome, the secondary outcomes, and other variables assessed at this time points – are described below.

#### Primary outcome

Depressive symptom severity observed at the end of treatment will be considered the primary outcome. Depressive symptom severity will be assessed using the PHQ-9 [23, 24]. This instrument has been previously described in the recruitment section.

#### Secondary outcomes

- Pain intensity. The Composed Pain Intensity Index [26] will be used to assess pain intensity. Patients will be asked to rate their lowest, medium, and strongest pain during the previous week, as well as their current pain, on a scale ranging from 0 (not at all) to 10 (extremely painful). The mean of these four scores will be calculated to obtain the average pain intensity. These scales have been shown to be valid and reliable and to be sensitive to treatment effects in several studies [26].
- Fibromyalgia impact. The Fibromyalgia Impact Questionnaire Revised (FIQ-R) [27] will be used to assess this variable. It comprises 21 items with response options ranging from 0 to 10, which are organized in three subscales: The Functioning Scale (nine items), the Fibromyalgia Severity Symptoms Scale (10 items), and the General Impact subscale (two items). The Functioning Scale assesses difficulties to perform several activities during the last week. The Symptoms Scale includes items assessing the severity of 10 symptoms that frequently affect patients with fibromyalgia (e.g., memory problems, body stiffness). Finally, the General Impact subscale assess the overall impact of fibromyalgia on functioning and symptom severity. A total fibromyalgia impact score (range 0–100) can also be calculated by adding the score of the Function subscale divided by 3, plus the score of the severity symptoms domain divided by 2, plus the scores of the General Impact subscale. The Chilean version of the FIQ-R has shown adequate psychometric properties in a sample of women with fibromyalgia (*N* = 94) (unpublished results).
- Pain catastrophizing. The Pain Catastrophizing Scale (PCS) [28, 29] will be used. The PCS is one of the most frequently employed scales to assess pain catastrophizing. It comprises 13 items regarding catastrophic pain-related thoughts and emotions that are organized in three subscales: rumination, magnification, and helplessness. Each item has five response options going from 0 (nothing) to 4 (all the time). The validity and reliability of the PCS has been broadly described in clinical and non-clinical samples. The psychometric properties of the Chilean version of the PCS has shown to be adequate in a sample of Chilean women with fibromyalgia (*N* = 76) (unpublished results).
- Pain hypervigilance. The Pain Vigilance and Awareness Questionnaire (PVAQ) [30] will be used to assess pain-related anxiety, specifically its cognitive aspects (namely, pain hypervigilance). It comprises nine items organized in two subscales: Active vigilance and active awareness. This questionnaire has excellent internal consistency (Cronbach alpha values between .82 and .92) and it has been shown to be valid [31]. The psychometric properties of this questionnaire have shown to be adequate in a sample of Chilean women with fibromyalgia (*N* = 119) (unpublished results).
- Self-reported physical health symptoms. The Patient Health Questionnaire (PHQ-15) [31] will be used to assess physical health symptoms. It comprises 15 items inquiring about somatic symptoms, which account for more than 90% of the symptoms reported in outpatient settings. Respondents rate the severity of each symptom on a 3-point scale (i.e., 0 = Not bothered at all, 1 = Bothered a little, 2 = Bothered a lot). The PHQ-15 enables classification of participants into four categories according to the reported severity of their symptoms: minimal (scores = 0–4), low (scores = 5–9), medium (scores = 10–14), and high (scores = 15–30). The PHQ-15 has excellent internal reliability and adequate convergent validity [31].
- Environmental reward. The Reward Probability Index, RPI [32] will be used to assess this variable. It comprises 20 items which assess access to environmental reward and are organized in two factors: Reward Probability and Environmental Suppressors, with strong internal consistency (α = .90). The instrument has been shown to have adequate convergent and discriminant validity [32].
- Activation. The Activation subscale of the Behavioral Activation for Depression Scale (BADS) [33] will be used to assess this variable. The BADS [33] is an instrument aimed at measuring changes in avoidance and activation over the course of the BA therapy. Its Spanish version showed adequate psychometric properties [34]. In a recent study conducted with Latino participants with depression, a BA intervention was found to produce not only a greater decrease in depressive symptoms than supporting counseling, but also increased activation and environmental reward [35]. The Cronbach alpha for the Activation subscale is .81.

#### Other variables

At each outcome assessment point, we will ask participants whether they have started any other treatment for pain or depression during the study, as concurrent therapies are not allowed. Furthermore, information about health behaviors and activities, such as tobacco or marihuana smoking, alcohol consumption, exercising, and participation in recreational activities, will also be recorded.

#### Patient’s treatment satisfaction

After completing the intervention participant’s treatment satisfaction will be assessed using the Client Satisfaction Questionnaire-8 (CSQ-8) [36]. This instrument is comprised of eight items. Its scores range from 1 to 32. Its Spanish version has adequate psychometric properties [37]. In addition, a modified version of the Verona Service Satisfaction Scale (VSSS) [38], comprising 14 items assessing satisfaction with the therapists and other aspects of treatment will be administered.

#### Adherence

Attendance to treatment sessions will be tracked as a measure of adherence. Treatment sessions will be video recorded and classified according to the phase of treatment (beginning, half, or end of treatment) in order to assess therapist adherence to the clinical guide. A research assistant will randomly select 20% of these sessions, which will be analyzed according to the adherence checklist included in the BA manual by two psychologists trained in BA.

### Data management

Collected paper questionnaires will be kept locked in a cabin within the office of the principal investigator, separated from the signed informed consent documents. To protect confidentiality, the questionnaires will not include personal identification information and they will be linked to each participant by a code randomly created by a computer. A file with the link between participants and their codes will be password-protected and kept in a computer in the office of the principal investigator. A research assistant who will be blind to randomization will enter the information into a password-protected database that will also be kept in a computer in the office of the principal investigator. Another blind research assistant will double check 10% of the data entered for data quality purposes. A data monitoring committee has not been deemed necessary because all participants receive UC and the intervention is not expected to lead to serious adverse events. However, adverse events will be monitored as part of the outcome assessments, including assessment of suicidal ideation. In case of imminent suicide risk, the participant’s physician and a close relative (who will be designated by the participant during the informed consent process) will be contacted and given instructions to take care of the patient, including taking them to an emergency psychiatric unit. If the designated family member is not available at the time, the patient will be taken to an emergency psychiatric unit by a research assistant.

### Analytic plan

#### Data analyses

In order to test whether there are differences between the two groups in the sociodemographic and clinical characteristics assessed at baseline, Student’s *t* tests will be performed for continuous variables and chi-square tests for categorical variables. If there are small or empty cells in the categorical tests, the chi-square test will be replaced by a Fisher’s Exact Test. Those variables in which statistically significant differences are found will be introduced in the analyses as covariates.

In order to examine differences between groups in the reduction of the primary and secondary outcome, HLM will be performed using MPlus. This analysis involves two levels of analysis: within-subject (level 1, which analyzes the change of subjects over time) and between-subject (level 2, through which the effect of treatment is examined). Repeated measures of symptoms are nested in each subject, and the analysis allows for estimating a change parameter along repeated measurements. The analysis tests whether the change parameter varies according to the treatment group, and other covariates. HLM allows one to consider in the analyses all participants (including those who drop out from the study). Therefore, these analyses will be conducted according to the principle of intention to treat. Finally, in order to test whether the decreases found after the follow-up in the pain-related variables are mediated by the decrease in depressive symptom severity, mediation analysis with bootstrapping will be conducted [39].

#### Analysis of the clinical relevance of the outcome changes

To examine whether the changes observed in depressive symptoms are clinically significant, the percentage of participants whose depression remits according to the MINI at the end of treatment will be compared between the two groups, using two Cochran-Mantel-Haenszel tests (one for the results obtained after the intervention and another for the results obtained after follow-up). In addition, changes in depressive symptoms in terms of response to treatment will be analyzed. That is, we will compare the percentage of patients who experience a reduction of at least 50% in the severity of depressive symptoms at the end of the treatment with the percentage of patients who do not respond to treatment [8] using the Cochran-Mantel-Haenszel test. To examine whether changes in pain intensity are clinically significant, we will compare the percentage of women responding to treatment in terms of the intensity of pain at the end of treatment with the percentage of women not responding to treatment using the Cochran-Mantel-Haenszel test. Treatment response will be defined as reductions of at least 2 units on the pain scale from 0 to 10, as this is the criteria usually employed for clinically significant pain improvement [40].

#### Missing data

Participants who are retained throughout the follow-up will be compared with those who drop out from the study in terms of socio-demographic and clinical variables. Any significant difference between groups will be considered in the interpretation of results. As mentioned before, HLM will be conducted for group comparison. These analyses will use maximum likelihood estimation with robust standard errors for variables that are not normally distributed. This estimator has been shown to be robust in estimating missing data [41].

## Discussion

Despite preliminary evidence suggesting that BA could be particularly suitable for treating depression among patients with fibromyalgia – as it addresses factors that seem to play a key role in the etiology of both disorders [6, 11–13] – to our knowledge, no studies have been conducted to examine the efficacy of this intervention in reducing depressive symptoms among these patients. This is important, as fibromyalgia and depression are highly coexistent and the interaction of both disorders leads to a worse prognosis. This is the first study in which the efficacy of BA in patients with fibromyalgia and major depression will be examined.

There are some limitations to the study design. First, all the instruments employed are self-report scales. Second, the follow-up assessment will take place at 3 months and studies with longer follow-up must be conducted in order to know whether the effects of the treatment are maintained in the long term. Third, participants under treatment with an antidepressant other than duloxetine will be excluded and, therefore, our results may not generalize to these participants. Nonetheless, requiring all subjects to be receiving duloxetine makes the sample more homogeneous, which is important in randomized clinical trials. Furthermore, as all participants will present with depression despite being under treatment with duloxetine, they will be considered unresponsive to duloxetine treatment. Thus, if we find that adding BA to UC reduces depressive symptoms, this can be interpreted as evidence that BA may be useful for patients who are refractory to duloxetine treatment. Finally, complete blinding of subjects is impossible given the type of intervention and fibromyalgia. Nevertheless, in this study all participants will receive the BA intervention at some point. The experimental and control groups will only differ in that the former will receive treatment before participants in the control group. As such, all participants will know that they will have to wait to receive the intervention, but they will not be given precise information about how long they will have to wait to receive it. Thus, they will not be aware of whether they are in the experimental group or in the control group. Finally, patients are known to be susceptible to placebo effect regarding pain [42], so future studies in which the effect of adding BA to UC is compared with the effect of adding an active intervention for depression to UC are still needed.

The results of this study should contribute to facilitate the integrated treatment of fibromyalgia and depression, and to reduce the burden to which health systems are exposed due to the lack of effective therapeutic strategies to treat these co-occurring conditions. In addition, we expect that our study contributes to the dissemination of evidence-based treatment, such as BA, among primary care professionals in Latin American countries. Our findings will also help to better understand the relationship between depression and pain. Finally, because BA will be applied in a group setting, our results will add support to the efficacy of BA in group format.

## Trial status

Protocol approved by the Ethic Committee CÓDIGO 15–291 (Eficacia de la terapia de activación conductual para pacientes con dolor crónico: ensayo clínico randomizado, Fonis regular 305). Version N° 3, June 2017. Trial registration: this study was registered at ClinicalTrials.gov under the name ¨Testing Interventions for Patients with Fibromyalgia and Depression¨ (Identifier: NCT03207828) on 5 July 2017 (last update posted 21 September 2017). ClinicalTrials.gov is an International Committee of Medical Journal Editors (ICMJE)-approved registry, so it is included in the World Health Organization Trial Registration Data Set.

The recruitment began on 9 September 2017 and is expected to be completed by 20 December 2018.

## Additional file

Additional file 1: Standard Protocol Items: Recommendations for Interventional Trials (SPIRIT) 2013 Checklist: recommended items to address in a clinical trial protocol and related documents. (DOC 123 kb)

## Abbreviations

BA: Behavioral activation
BADS: Activation subscale of the Behavioral Activation for Depression Scale
CSQ-8: Client Satisfaction Questionnaire-8
FIQ-R: Fibromyalgia Impact Questionnaire
FSQ: Fibromyalgia Survey Questionnaire
HLM: Hierarchical Linear Model
MINI: Mini International Neuropsychiatric Interview
MMSE: Mini-Mental State Examination
PCS: Pain Catastrophizing Scale
PHQ-15: Patient Health Questionnaire-15
PHQ-9: Patient Health Questionnaire-9
PVAQ: Pain Vigilance and Awareness Questionnaire
RPI: Reward Probability Index
UC: Usual care
VSSS: Verona Service Satisfaction Scale
